# Is left-sided involvement of hepatocellular carcinoma an important preoperative predictive factor of poor outcome?

**DOI:** 10.1186/s12957-020-02100-6

**Published:** 2020-12-03

**Authors:** Yuhki Sakuraoka, Keiichi Kubota, Genki Tanaka, Takayuki Shimizu, Kazuma Tago, Kyung Hwa Park, Takatsugu Matsumoto, Takayuki Shiraki, Shozo Mori, Yukihiro Iso, Taku Aoki

**Affiliations:** Second Department of Surgery, Dokkyo Medical University, 880 Kitakobayashi, Mibu, Tochigi, 321-0293 Japan

**Keywords:** Hepatocellular carcinoma, Liver surgery

## Abstract

**Background:**

The tumor location is the most simple clinical factor and important in liver surgery to make surgical procedure. However, no previous study has investigated the prognostic differences and clinical features of hepatocellular carcinoma showing specific laterality. This study is the first report to focus on the laterality and aimed to lead to more simple and useful predictive factor rather than recent complicated predictive models.

**Methods:**

Patients who underwent liver resection for the first time for single tumors located within each lobe between 2000 and 2018 were enrolled. We divided them into two groups based on tumor location: a right-sided group and a left-sided group. Univariable and multivariable analyses were performed to assess survival differences in relation to several other factors.

**Results:**

There were 595 eligible patients; the 5-year survival rates and disease-free survival rates were 49.5% and 19.1% in the left-sided group and 55.6% and 24.5% in the right-sided group, respectively (*p* = 0.026). Statistical analyses revealed that the following preoperative prognostic factors were independently significant (*p* < 0.05) in the left-sided group: indocyanine green retention rate at 15 min, alpha fetoprotein, protein induced by vitamin K absence or antagonists-II level, and larger tumor size.

**Conclusion:**

The left-sided group had a poorer prognosis than the right-sided group. A left-sided tumor location is a significant preoperative factor predictive of poor outcome in patients with hepatocellular carcinoma.

## Background

Recently, there have been several types of predictive factors in liver surgery. Some of them are complicated and difficult to perform in daily clinical settings. We explored more simple predictive factors in order to easier project the patient outcomes. In this study, we focused on the tumor location. This is because it is easy and simple to detect. Although the tumor location is important in liver surgery to make surgical procedure, no previous study has investigated the prognostic differences and clinical features of hepatocellular carcinoma (HCC) showing specific laterality.

In general, the organs located on the left and right sides of the body show some specific anatomical features. For instance, the right lung has three lobes, whereas the left lung consists of two lobes. Moreover, the hemispheres of the brain show unique differences in terms of function. The right lobe of the liver is divided into four segments, while the left lobe consists of three segments [1, 2]. Previous studies have revealed that there are also some differences in the malignant characteristics and prognosis of tumors located in left- and right-sided organs.

Renal cell carcinoma (RCC) located on the left with tumor thrombus does not have a worse prognosis than RCC located on the right [3]. In the thyroid, Gessl et al. showed that a larger proportion of both benign and malignant tumors tended to affect the right lobe [4]. However, no study has attempted to investigate differences in features between the right and left hepatic lobes. In the present study, we aimed to explore the clinical features of laterality in HCCs and consider the probability of the predictable power for the prognosis in preoperative factors.

## Materials and methods

This retrospective single-center study was conducted between April 2000 and 2018. All patients undergoing liver resection for the first time for a single tumor located within the right lobe or left lobe, and who had been diagnosed pathologically as having HCC were enrolled. Essentially, the left lobe of the liver is divided to the left by the middle hepatic vein whereas the right lobe is defined as the portion of the liver to the right side of the middle hepatic vein [1, 2]. On the basis of this, as the definition showed below, we divided the eligible patients into two groups based on the location of the tumor in the liver: a right-sided group (RSG), whose tumors were located within the right lobe, and a left-sided group (LSG), whose tumors were located within the left lobe. We excluded the tumor which had expanded to both lobes, multiple HCCs, or lesions in the caudate lobe.

All data were collected retrospectively by using medical chart review. We investigated the gender, ages, and physical features of the patients in both groups, including pathological findings in the liver. The collected clinical data included the preoperative retention rate of indocyanine green at 15 min (ICGR15), the alpha fetoprotein (AFP) level, and the level of protein induced by vitamin K absence or antagonists-II (PIVKA2). In principle, at our center, anatomical (AR) or non-anatomical (NAR) liver resection is performed in accordance with the Makuuchi criteria [5–8]. Therefore, in both groups of patients, the types of operations were classified for comparison. In this study, we excluded laparoscopic surgery and liver transplant throughout our observation periods.

The operation time and total amount of blood loss were recorded. Based on pathological findings, the differences in tumor size and total tumor volume were recorded, and we investigated whether or not the tumors had invaded the portal vein, hepatic vein, or bile duct. Specifically, we recorded the incidence of hepatic vein invasion or thrombus (vv), portal vein invasion or thrombus (vp), hepatic artery invasion or thrombus (va), or biliary duct or thrombus (b). Moreover, the tumor’s growth pattern was evaluated (expansive growth (EG) or invasive growth (IG)), and the data were compared between the two groups.

Patients visited the hospital once a month for the first 12 months after surgery and at 2- to 3-month intervals thereafter. Tumor markers, including AFP and PIVKA2, were examined at each visit. Patients were monitored by contrast-enhanced computed tomography of the chest and abdomen at 3-month intervals for the first 12 months and at 4-month intervals thereafter. During the follow-up, re-hepatectomy was performed for the patients who were detected under three numbers of recurrent lesions. Patients who were not eligible as the curative operation were planned to perform chemotherapy, transcatheter arterial chemoembolization (TACE), or best supportive care. We excluded the patients who were lost follow-up from this study. We also examined postoperative complications in terms of the number of patients whose postoperative ascites was more than 1000 ml and the figure for patients with pleural effusion requiring thoracentesis. We investigated patients’ prognosis using Kaplan-Meier analysis and log-rank test for comparison of overall survival (OS) and relapse-free survival (RFS). Secondly, we examined the cumulative number of patients with hematogenous metastatic lesions and recurrent lesions in the liver after curative resection. The date of recurrence was defined as the first examination date when recurrence was observed by imaging modalities, including ultrasound, computed tomography (CT), magnetic resonance imaging (MRI), and angiography. Furthermore, univariate analysis was performed using the Cox proportional hazards model to detect the clinical characteristics that were correlated with OS and RFS. Then, multivariate analysis was carried out to evaluate statistically any differences in laterality among several preoperative factors for which significant differences were demonstrated by univariate analysis.

## Definition

We defined RSG as the single tumor of HCC that existed at the right side of the middle hepatic vein in the liver. Meanwhile, LSG was the single tumor of HCC that existed at the left side of the middle hepatic vein [1, 2]. More specifically, the tumor of segments 5, 6, 7, and 8 in the right lobe of the liver was considered as RSG and segments 2, 3, and 4 in the left lobe of the liver was LSG.

## Statistical analysis

All data were analyzed using the SPSS statistical software package (Ver 25.0; SPSS Inc., Chicago, IL, USA). Survival was estimated using the Kaplan-Meier method, and survival estimates were compared using the log-rank test. Data were censored on July 1, 2018. Patients who were lost to follow-up were excluded from this study to lead to the accurate statistical results. Patients who were alive on July 1, 2018, were censored for OS analysis. Relapse-free survival was calculated from the data at diagnosis until the date of disease recurrence or HCC-related deaths. OS was calculated from the data at diagnosis to the date of HCC-related deaths and OS outcome contained only HCC-related deaths. Continuous variables were presented as the median and range and were compared using the Mann-Whitney *U* test. Categorical variables were compared using Fisher’s exact test, and continuous variables were examined using Student’s *t* test. Univariable analyses were performed to identify the significant distinctive prognostic factors of the LSG vs the RSG using the Cox regression model. Differences at *P* < 0.05 were considered statistically significant. The multivariable model was performed in preoperative factors and used to compare other predictive factors related to HCC prognosis that were shown to be significantly different by univariable analysis. Regression models were used to obtain hazard ratios and their confidence intervals [9, 10], and the proportional hazard assumption was checked for all variables. In order to evaluate interaction effect, variance, and confounding, analysis of variance (ANOVA), Brown-Forsythe, and Bartlett’s tests were additionally performed.

## Results

### Patient background

During the study period, 947 patients were diagnosed pathologically as having HCC at Dokkyo Medical University. The tumor was situated in the right lobe in 406 patients, whereas it was situated in the left lobe in 189. Four hundred and two patients were considered ineligible for the study because the tumor location was beyond the middle hepatic veins or multiple tumors were located in both lobes. The median age of the patients was 69 years, and there were 471 men and 124 women. The median body mass index (BMI) was 23.

With regard to disease etiology in relation to viral infection, 119 patients were positive for hepatitis B virus (HBV+), 191 were positive for hepatitis C virus (HCV+), 195 were both HBV+ and HCV+, and 91 had neither HBV+ nor HCV+. There were 15 patients with normal liver, 279 with liver cirrhosis, and 301 with chronic hepatitis. The median tumor size was 2.8 cm. AR was performed for 334 of the patients, and NAR for 261 (Table 1).

**Table 1:** Patients’ background

Characteristics	Total patients (***n*** = 595)
**Age (year)**	21–84 (median 69)
**Gender (male/female)**	471/124
**BMI**	15.3–39.2 (median 23)
**Disease etiology**	
HBV+	119
HCV+	191
HBV+ HCV+	195
HBV− HCV−	91
**Background of liver**	
Normal liver	15
Chronic hepatitis	301
Liver cirrhosis	279
**Tumor size (cm)**	0.6–40 (median 2.8)
**Surgical procedure**	
AR	334
NAR	261

In this study, we defined HBV positivity as positivity for any antigens and HCV positivity as positivity for antibody against HCV. Liver fibrosis grade was diagnosed on the basis of the Inuyama criteria, which classifies the background liver from f0 to f4

*BMI* body mass index, *HBV* hepatitis B virus, *HCV* hepatitis C virus, *AR* anatomical resection, *NAR* non-anatomical resection

### Univariate analysis of LSG and RSG

The patients’ background factors revealed significant differences in operation time between the two groups. That in the RSG was 285 min and that in the LSG was 250 min. However, there were no inter-group differences in clinical laboratory data or tumor markers. The amount of blood loss and the numbers of AR and NAR also showed no significant inter-group differences. With regard to pathological findings, the median maximum tumor diameter was 28 mm in the RSG and 25 mm in the LSG (*p* = 0.61). The total tumor volume in the RSG was 11.5 cm^3^ and that in the LSG was 9.8 cm^3^. Furthermore, 66 patients had more than 1000 ml of ascites in the RSG and 33 did so in the LSG (*p* = 0.65). There were 89 cases of pleural effusion in the RSG and 9 in the LSG, the difference being significant (*p* = 0.02). With regard to the pattern of recurrence, there were significant differences among the numbers of patients which had metastatic lesions in the lung, bone, or brain, being 29 in the RSG and 23 in the LSG (*p* = 0.044). There was no significant inter-group difference in the number of patients with liver metastasis (*p* = 0.64): 236 in the RSG and 114 in the LSG (Table 2).

**Table 2:** Variables in the RGS and LGS

Characteristic	RGS (*n* = 406)	LGS (*n* = 189)	*p* value
**Background**			
Age	68	69	0.12
Gender (male/female)	326 / 80	145 / 44	
BMI	23.9	24.1	0.89
**Preoperative laboratory data**			
AST	36 (10–158)	41 (24–114)	0.58
ALT	31 (3–183)	39 (13–172)	0.51
Alb	3.5 (1.6–4.9)	3.6 (2.1–4.8)	0.65
ICGR15	15 (1–74)	17.7 (4–48)	0.61
PT%	83(28–120)	84 (0.1–20)	0.87
AFP	21	15	0.11
PIVKA2	46	45	0.86
**Operative findings**			
Operation time (min)	285	250	**0.0024**
Bleeding (ml)	497	468	0.39
**Pathological findings**			
Tumor size (mm)	28	25	0.61
Total tumor volume	11.5	9.8	0.62
vv + (*n*=)	16	10	0.46
vp + (*n*=)	108	52	0.83
va + (*n*=)	2	2	0.43
b + (*n*=)	7	7	0.16
IG	39	24	0.26
EG	367	165	0.18
**Postoperative course**			
Ascites > 1000 (*n*=)	66	33	0.65
Pleural effusion (*n*=)	89	9	**0.02**
**Mode of operation**			
**Anatomical resection**	229	105	0.87
Lobectomy	12	28	< 0.0001
Sectionectomy	34	43	< 0.0001
Segmentectomy	142	※	–
**Non-anatomical resection**	–	–	–
Partial resection	177	84	0.83
**Pattern of recurrence**			
**Hematogenous metastasis**	29	23	**0.044**
**Intra hepatic metastasis**	236	114	0.64
Local recurrence	79	32	0.47
Distant inside the liver	157	82	0.26

Sectionectomy was defined as lateral, median, anterior, and posterior hepatic resection. Segmentectomy means segment 5, 6, 7, and 8 resection. ^※^Because segment 4 resection was considered as median hepatic resection, segmentectomy in LGS was not applicable as shown. As for pattern of recurrence, local recurrence was defined as recurrent tumor located within the right lobe in RGS or the left lobe in LGS. This included single and multiple recurrent tumors

*AST* aspartate aminotransferase, *ALT* alanine aminotransferase, *Alb* albumin, *ICGR15* indocyanine green retention rate at 15 min, *PT%* prothrombin time and international normalized ratio, *AFP* alpha fetoprotein, *PIVKA2* protein induced by vitamin K absence or antagonists-II, *vv* hepatic vein invasion or thrombus, *vp* portal vein invasion or thrombus, *va* hepatic artery invasion or thrombus, *b* bile duct invasion or thrombus, *EG* expansive growth, *IG* invasive growth

### Overall survival and relapse-free survival

**Fig. 1:**
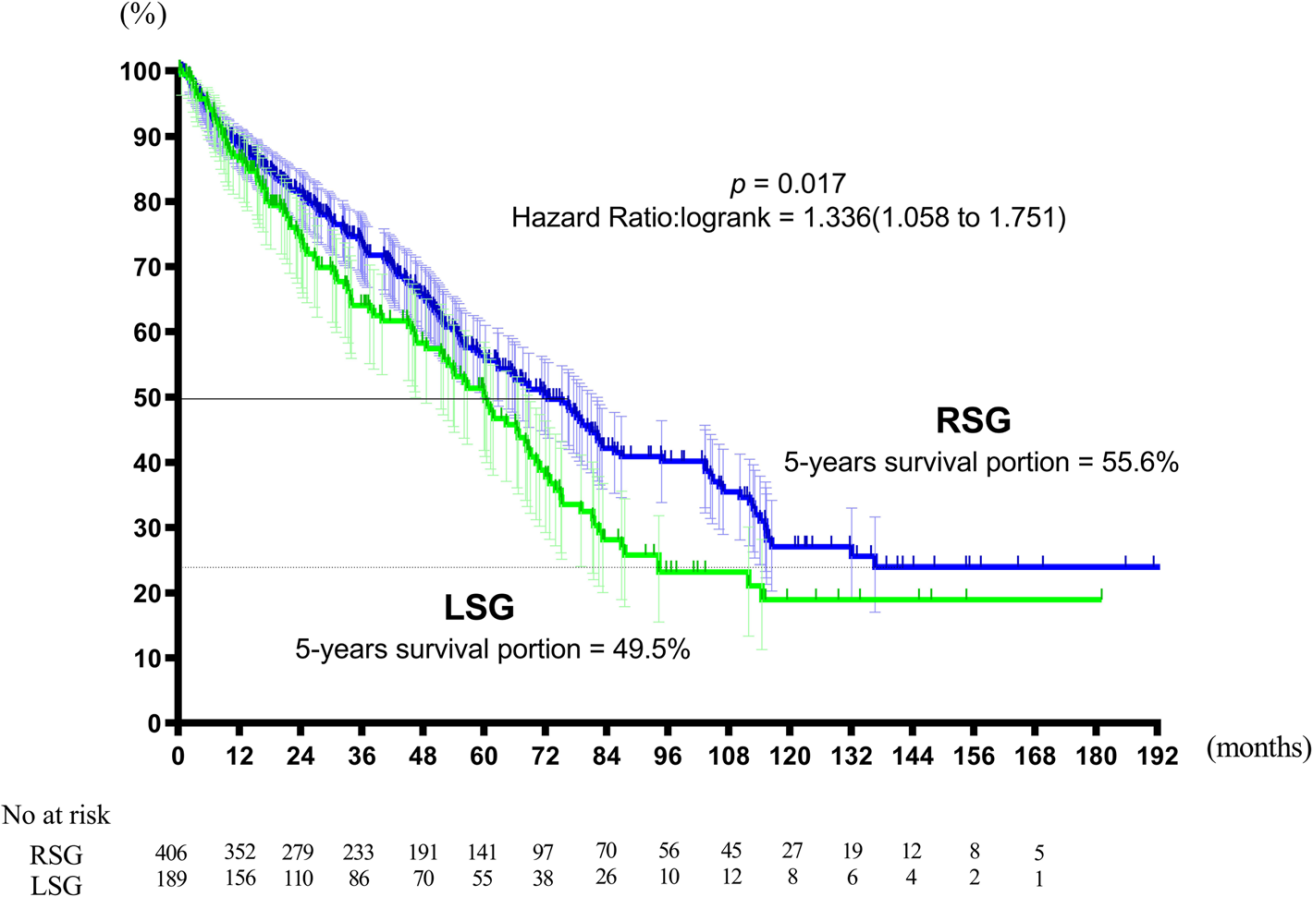
Overall survival. The median OS duration was 72.6 months in the RSG and 60.2 months in the LSG (*p* = 0.0017; hazard ratio 1.336; 95%Cl 1.058–1.751). OS was significantly shorter in the LSG. The 5-year cumulative survival rates were 49.5% in the LSG and 55.6% in the RSG (*p* = 0.017)

**Fig. 2:**
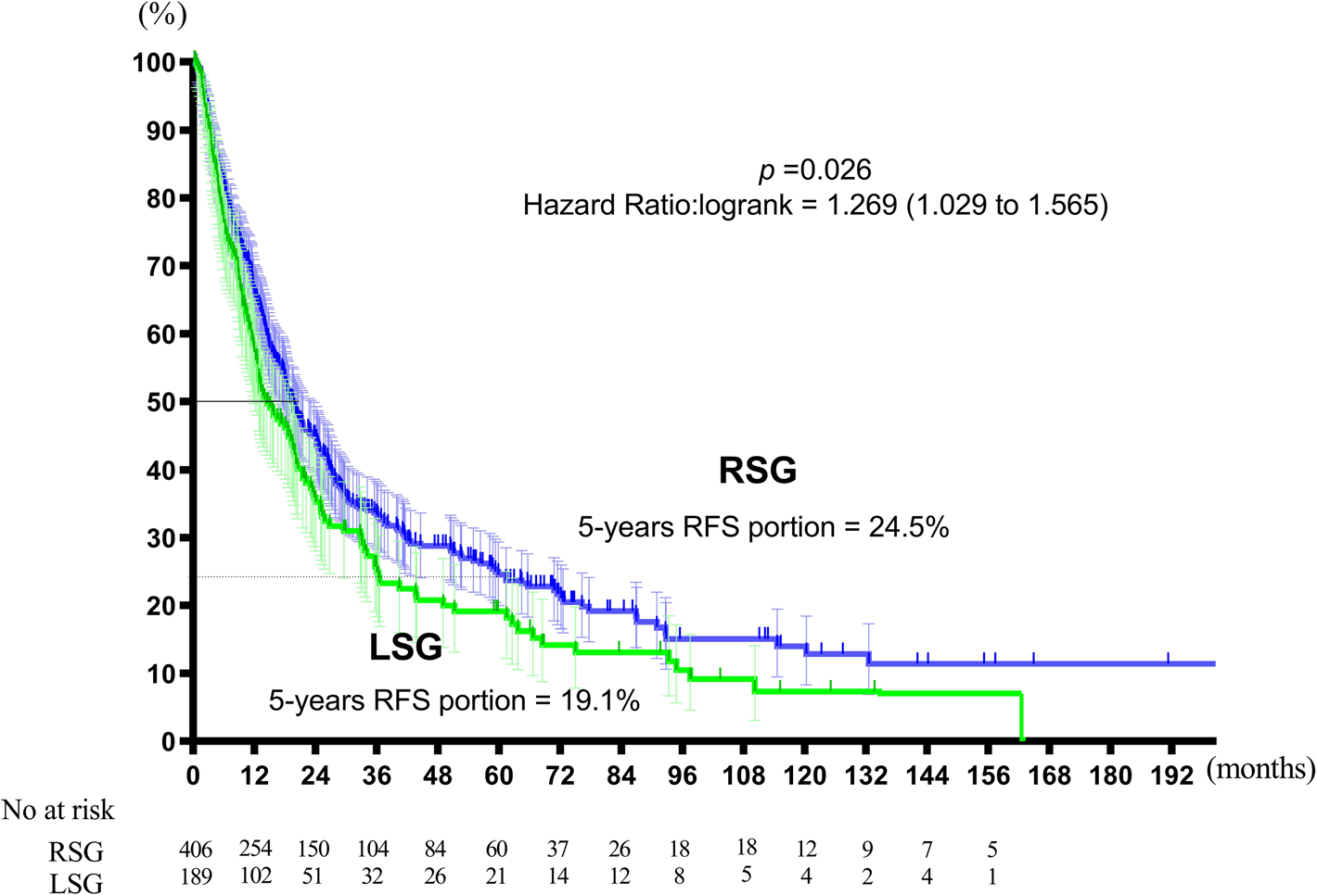
Relapse-free survival. The RFS was 19.6 months for the RSG and 14.5 months for the LSG (*p* = 0.0401; hazard ratio 1.269; 95%Cl 1.029–1.565). RFS was significantly shorter in the LSG. The 5-year cumulative disease-free survival rates was 19.1% in the LSG and 24.5% in the RSG (*p* = 0.026)

The median OS duration was 72.6 months in the RSG and 60.2 months in the LSG (*p* = 0.0017; hazard ratio 1.336; 95%Cl 1.058–1.751). The RFS was 19.6 months for the RSG and 14.5 months for the LSG (*p* = 0.0401; hazard ratio 1.269; 95%Cl 1.029–1.565). Both OS and RFS were significantly shorter in the LSG. The 5-year cumulative survival rates and cumulative disease-free survival rates were 49.5% and 19.1% in the LSG and 55.6% and 24.5% in the RSG, respectively (*p* = 0.026; Figs. 1 and 2).

### Univariable and multivariable analyses in overall survival

Univariable analyses of OS revealed that in the LSG the following factors were prognostically significant: resection type, ICGR15, AFP, PIVKA2, tumor size, operation time, blood loss, HBV, hepatic vein invasion, and portal vein invasion. On the basis of the results of log-minus-log plot ANOVA, Brown-Forsythe, and Bartlett’s tests, preoperative significant predictive factors including LSG, ICGR15, AFP, PIVKA2, tumor size, and HBV did not have interaction effect and confounders, and proportional hazard was confirmed. Multivariable analysis of preoperative factors that were prognostically significant in the univariable analyses showed that the LSG, ICGR15, AFP, and tumor size were significant. More specifically, in terms of OS, the results revealed that the LSG had a significant association with (presence/absence) (HR, 1.371; 95% CI 1.073–1.752; *p* = 0.012), ICGR (HR, 1.018; 95% CI 1.007–1.030; *p* = 0.002), AFP (< 13, > 13.1) (HR, 1.777; 95% CI 1.384–2.282; *p* < 0.001), and tumor size (< 5 cm, > 5.1 cm) (HR, 1.949; 95% CI 1.470–2.582; *p* < 0.001) (Table 3).

**Table 3:** Associations between cumulative death proportions and clinical factors demonstrated by univariable and multivariable analyses

		Univariable analysis					Multivariable analysis (only preoperative factors)				
		HR	95% CI			*p* value	HR	95% CI			*p* value^a^
Age	Continuous	1.018	1.004–1.031			**0.010**	1.018	1.002–1.033			**0.019**
Sex	Female	Ref					Ref				
	Male	0.844	0.642–1.109			0.220	1.049	0.785–1.402			0.745
Location	RGS	Ref					Ref				
	LGS	1.34	1.055–1.702			**0.018**	1.371	1.073–1.752			**0.012**
Resection type	Anatomical resection	Ref					Ref				
	Non-anatomical resection	1.407	1.119–1.768			**0.003**	–	–	–	–	–
ICGR	Continuous	1.019	1.008–1.03			**0.001**	1.018	1.007–1.030			**0.002**
AFP, ng/mL	< 13	Ref									
	> 13.1	1.804	1.413–2.302			**< 0.001**	1.777	1.384–2.282			**< 0.001**
PIVKA2, ng/mL	< 40	Ref									
	> 41	1.597	1.267–2.012			**< 0.001**	1.256	0.972–1.623			0.082
Tumor size, cm	< 5	Ref									
	> 5.1	2.134	1.647–2.766			**< 0.001**	1.949	1.470–2.582			**< 0.001**
Operative time, min	< 270	Ref		–							
	> 271	1.708	1.350–2.162			**< 0.001**	–	–	–	–	–
Blood loss, ml	< 300	Ref		–							
	> 301	1.590	1.216–2.079			**0.001**	–	–	–	–	–
HBV	Negative	Ref		–							
	Positive	0.697	0.487–0.998			**0.049**	0.943	0.635–1.400			0.771
HCV	Negative	Ref		–							
	Positive	1.083	0.850–1.380			0.518	–	–	–	–	–
Hepatic vein invasion	Negative	Ref		–							
	Positive	2.180	1.350–3.518			**0.001**	–	–	–	–	–
Portal vein invasion	Negative	Ref		–							
	Positive	1.705	1.340–2.171			**< 0.001**	–	–	–	–	–

*HR* hazard ratios, *95%CI* 95% confidence interval

^a^Input criteria for multiple analysis were preoperative factors with *p* < 0.05 in univariate analysis with age and sex

### Univariable and multivariable analyses in relapse-free survival

Univariable analysis of RFS revealed that in the LSG the following factors were prognostically significant: resection type, ICGR15, AFP, PIVKA2, tumor size, operation time, blood loss, HBV, hepatic vein invasion, and portal vein invasion. In preoperative factors, multivariable analyses of that were prognostically significant, and the univariable analysis showed that the LSG, ICGR15, AFP, PIVKA2, and tumor size were significant. LSG was one of the significant preoperative factors similar to the results of OS (Table 4).

**Table 4:** Associations between relapse-free survival proportions and clinical factors demonstrated by univariable and multivariable analyses

		Univariable analysis					Multivariable analysis (only preoperative factors)				
		HR	95% CI			*p* value	HR	95% CI			*p* value^a^
Age	Continuous	1.016	1.012–1.034			0.36	1.011	1.005–1.017			0.062
Sex	Female	Ref					Ref				
	Male	0.892	0.542–1.076			0.407	1.324	1.197–1.451			**0.028**
Location	RGS	Ref					Ref				
	LGS	1.269	1.029–1.565			**0.026**	1.208	1.104–1.312			**0.048**
Resection type	Anatomical resection	Ref									
	Non-anatomical resection	1.372	1.024–1.838			**0.022**	–	–	–	–	–
ICGR	Continuous	1.021	1.018–1.032			**< 0.001**	1.011	0.007–1.022			**0.019**
AFP, ng/mL	< 13	Ref					Ref				
	> 13.1	1.631	1.416–1.879			**< 0.001**	1.555	1.114–1.654			**< 0.001**
PIVKA2, ng/mL	< 40	Ref					Ref				
	> 41	1.475	1.28–1.699			**< 0.001**	1.252	1.146–1.358			**0.035**
Tumor size, cm	< 5	Ref					Ref				
	> 5.1	2.221	1.745–2.783			**< 0.001**	1.897	1.773–2.021			**< 0.001**
Operative time, min	< 270	Ref		–							
	> 271	1.558	1.354–1.792			**< 0.001**	–	–	–	–	–
Blood loss, ml	< 300	Ref		–							
	> 301	1.429	1.233–1.657			**< 0.001**	–	–	–	–	–
HBV	Negative	Ref		–			Ref				
	Positive	1.431	1.224–1.672			**< 0.001**	0.821	0.656–0.986			0.231
HCV	Negative	Ref		–							
	Positive	1.155	0.997–1.351			0.055	–	–	–	–	–
Hepatic vein invasion	Negative	Ref		–							
	Positive	1.902	1.264–2.861			**< 0.001**	–	–	–	–	–
Portal vein invasion	Negative	Ref		–							
	Positive	1.456	1.231–1.724			**< 0.001**	–	–	–	–	–

*HR* hazard ratios, *95%CI* 95% confidence interval

^a^Input criteria for multiple analysis were preoperative factors with *p* < 0.05 in univariate analysis with age and sex

## Discussion

The results of this study revealed that left-sided HCC had a significantly poorer outcome and a higher rate of hematogenous recurrence than right-sided HCC. Multivariable analysis showed that the laterality was one of the significant preoperative predictable factors. In this study, especially for multivariable analysis, we focused on the preoperative factors and only included simple factors. This is our firm belief that preoperative simple factors only play the beneficial role in clinical oncology and are useful to predict the patient outcomes in clinical settings.

Previous studies of HCC have revealed that the presence of vascular invasion [11], higher levels of AFP and PIVKA2 [12], the presence of hepatitis [13], a larger tumor size [10, 14], higher ICG levels and rates of liver cirhosis [15], and a higher amount of intraoperative blood loss [16–18] are strong and independent predictors of outcome. Among them, our results revealed that ICG, AFP, PIVKA2, and tumor size had predictive prognostic power as the preoperative factors (Tables 3 and 4). Earlier studies have shown that the ability of the hepatocytes to take up ICG becomes rate limiting at very high concentrations, and ICG clearance (ICG-K) under these circumstances represents a sensitive measure of liver cell function [19]. Several reports revealed ICG can predict accurate liver function. In our department, we made surgical procedure on the basis of the level of ICT test. Anatomical resection, which had the better prognostic result as shown in Table 3, only undertook for the patients who had the lower level of ICG. We considered this is the reason why our results showed the higher level of ICG lead to the poor prognosis. Meanwhile, AFP and PIVKA2 are reliable markers to predict patient outcomes reflecting tumor biology. These markers are also useful to detect recurrence after curative resection [20]. In relation to tumor size, it is an important factor to estimate stage. Huang et al. reported that it is the most important determinant of RFS and OS in resected 230 primary-stage I HCC patients [21]. Moreover, Goh et al. also revealed that the tumor size is one of the independent prognostic factors of RFS for solitary HCC after LR in 560 HCC patients [22].

In addition to these well-known factors, one previous study found that left-sided partial liver resections were associated with poorer outcomes [23]. However, that study explored only partial resection in a small number of cases. In contrast, the present study was performed with a far larger number of patients and included several types of operations, although similar results and associations with left-sided tumor resection were found. Moreover, multivariate analysis revealed that left-sided resection was one of the significant predictive factors of OS when we focused on preoperative factors.

In general, the right lobe is larger than the left lobe. This partly accounts for the fact that a larger proportion of HCCs are located on the right side. Despite this, our results illustrate that HCCs located in the left lobe have a poor prognosis and show a higher frequency of hematogenous metastasis. We consider that there are two major reasons for this.

One possible reason for the higher HCC recurrence with left-sided resection is the larger size of the liver remnant. HCC develops according to the background of the underlying chronic liver disease, such as cirrhosis or viral hepatitis. Larger liver remnants such as those resulting from left-sided liver resection include larger amounts of liver tissue, with a risk of future HCC development. Furthermore, the present univariable analysis illustrated that a higher ICG retention rate contributed to poorer overall survival. This could possibly mean that remnant liver with a poorer tissue background might tend to have a higher frequency of recurrent HCC.

Another potential explanation could be the resection margin. Previous studies have revealed that the resection margin may be a vital factor that influences the recurrence rate [24–26]. As mentioned above, the left lobe of the liver is smaller than the right lobe. Thus, the margin after left-sided resection may be smaller than that after right-sided resection, thus leading to a poorer outcome.

To our knowledge, clinical outcomes of HCC patients have not been studied previously in relation to the surgical site in terms of left-sided versus right-sided resection. Our study is the first to explore differences in patient outcome according to tumor location. Nevertheless, this study had some limitations. It was retrospective in design, which limited our ability to obtain certain types of data. However, our primary outcome variable was HCC recurrence, and our primary predictor variable was left-sided versus right-sided resection, which are both objective and readily available data. Moreover, this study focused on preoperative factors in terms of prediction for prognosis. Laterality might not keep the statistical significance if we put all clinical factors randomly. Again, this is our firm belief that preoperative factors only have significance to project patient outcomes in clinical settings. For this reason, we put only preoperative factors which had significant difference in univariable analyses into multivariable analysis.

Other limitations were the single-center design and small sample size. The vast majority of our patients had viral hepatitis, and therefore, our results may not be generalizable to HCC patients with non-viral liver disease and those with non-alcoholic steatohepatitis (NASH) [27]. Additionally, all of the patients were Asian, which limits the application of our conclusions to other ethnic groups. However, our study included well-defined consecutive HCC patients undergoing hepatectomy at a university medical center.

## Conclusions

The LSG had a poorer outcome than the RSG. Our results showed that tumor laterality is a significant preoperative factor affecting the outcome of HCC. To our knowledge, this is the first study to have compared the prognosis of HCC between left-sided and right-sided resections. Our results show that laterality is an important preoperative variable predictive of outcome in patients with HCC. Clinicians need to be aware of this factor because laterality is a very basic variable that is easy to determine.

## Data Availability

We will provide all data and material which were used for this study in accordance with requirement from readers.

## Abbreviations

AFP: Alpha fetoprotein
AR: Anatomical resection
b: Bile duct invasion or thrombus
CT: Computed tomography
EG: Expansive growth
HBV: Hepatitis B virus
HCC: Hepatocellular carcinoma
HCV: Hepatitis C virus
IG: Invasive growth
ICGR15: Indocyanine green retention rate at 15 min
LSG: Left-sided group
MRI: Magnetic resonance imaging
NAR: Non-anatomical resection
OS: Overall survival
PIVKA2: Protein induced by vitamin K absence or antagonists-II
RCC: Renal cell carcinoma
RFS: Relapse-free survival
RSG: Right-sided group
TACE: Transcatheter arterial chemoembolization
TNM: Tumor, lymph node, metastasis staging system
vab: Hepatic artery invasion or thrombus
vp: Portal vein invasion or thrombus
vv: Hepatic vein invasion or thrombus
